# Simulated Biomechanical Analysis of Optimal Knee Alignment for Treating Medial Meniscus Posterior Root Tears

**DOI:** 10.1177/23259671251344944

**Published:** 2025-07-11

**Authors:** Takaaki Hiranaka, Adam Garry Redgrift, Yizhao Li, Larissa Michele Madia, Ryan Willing, Alan Getgood

**Affiliations:** * Fowler Kennedy Sport Medicine Clinic, Western University, London, Ontario, Canada; † Department of Orthopaedic Surgery, Section of Medicine, Graduate School of Medicine, Dentistry and Pharmaceutical Sciences, Okayama University, Okayama, Japan; ‡ Department of Mechanical and Materials Engineering, Western University, London, Ontario, Canada; § School of Biomedical Engineering, Western University, London, Ontario, Canada; ‖ Bone and Joint Institute, Western University, London, Ontario, Canada; ¶ Aspetar Orthopaedic and Sports Medicine Hospital, Doha, Qatar; Investigation performed at the Department of Mechanical and Materials Engineering, Western University, London, Ontario, Canada

**Keywords:** knee, medial meniscus posterior root tear, lower limb alignment, high tibial osteotomy, biomechanical study

## Abstract

**Background:**

Medial opening-wedge high tibial osteotomy (MOWHTO) is used to correct varus alignment; however, the optimal knee alignment during MOWHTO for medial meniscus posterior root tears (MMPRTs) remains unclear.

**Purpose:**

To determine the optimal biomechanical knee alignment for MMPRT treatment during MOWHTO.

**Study Design:**

Controlled laboratory study.

**Methods:**

This study used 10 fresh-frozen cadaveric legs from human donors (mean age, 61.3 years [range, 33-75 years]). A joint motion simulator assessed the weightbearing line (WBL) from 30% to 70% (0%: medial border; 100%: lateral border), simulating MOWHTO. Tibiofemoral peak contact pressure (PCP) and mean contact pressure (MCP) were measured using a pressure sensor under a 700-N load. MMPRTs were created via a femoral posterior approach and repaired with suture anchors. Measurements were taken in the intact, MMPRT, and repair conditions at alignments of 30% to 70% WBL, with neutral alignment defined as 50% WBL. Statistical analysis was performed using one-way analysis of variance with the Tukey post hoc test.

**Results:**

In the medial compartment, PCP was increased by 43% in the MMPRT condition compared with the intact condition at neutral alignment (*P* = .012). MCP was also significantly increased by 57% in the MMPRT condition compared with the intact condition (*P* = .006). At varus alignment, PCP and MCP increased in all conditions, with the largest statistically significant differences observed at 30% WBL (*P* = .002 and *P* < .001, respectively). PCP and MCP at neutral alignment in the intact condition were comparable with those at 60% to 65% WBL in the MMPRT condition and at 50% to 55% WBL in the repair condition. In the lateral compartment, PCP and MCP increased at valgus alignment, with no significant differences among conditions.

**Conclusion:**

MCP at neutral alignment in the intact condition was similar to that at 60% to 65% WBL in the MMPRT condition and at 50% to 55% WBL in the repair condition, indicating optimal biomechanical alignment targets for MOWHTO in patients with MMPRTs. 50–55% WBL corresponds to slight valgus alignment. Neutral alignment was not considered ideal in this context.

**Clinical Relevance:**

These findings provide biomechanical evidence to guide optimal knee alignment during MOWHTO for MMPRTs, potentially improving patient outcomes.

The posterior root of the medial meniscus is an important anatomic anchor that stabilizes the medial meniscus during knee motion, helping to distribute joint forces under loading. A medial meniscus posterior root tear (MMPRT) is closely linked to the progression of medial compartment osteoarthritis and subchondral insufficiency fractures primarily due to a loss of hoop tension within the meniscus when a compressive load is applied.^9,24^ Consequently, MMPRTs can lead to a 25% increase in peak contact pressure (PCP) in the medial compartment compared with an intact meniscus.^1^ Without appropriate treatment, 31% of MMPRT cases require total knee arthroplasty (TKA) at a mean time of 30 months.^25^

Current MMPRT repair techniques have been reported to restore normal knee function, reduce the risk of degenerative changes in the knee joint, and yield favorable clinical outcomes.^3,13^ MMPRT repair reduces the risk of the progression to TKA. Bernard et al^2^ reported a 0% rate of conversion to TKA after MMPRT repair compared with 60% and 26% for partial meniscectomy and nonoperative management, respectively, at a mean follow-up of 6 years. Similarly, Chung et al^8^ found a survival rate of 79.6% for repair versus 44.4% for meniscectomy at a minimum 10-year follow-up. Kim et al^21^ demonstrated lower rates of conversion to TKA with repair (22%) than with meniscectomy (56%) at a follow-up of approximately 4 years. These findings highlight the efficacy of MMPRT repair in reducing the rate of TKA over time.

Despite these positive outcomes, various prognostic factors are crucial for establishing the appropriate surgical indications for MMPRT repair. Varus alignment of the lower extremity (>5°) significantly affects outcomes after MMPRT repair. Moon et al^33^ found that patients with varus alignment >5° had lower functional scores and satisfaction than those with <5° varus alignment. Chung et al^6^ demonstrated that mechanical varus alignment >5° increased the likelihood of conversion to TKA (odds ratio, 1.5). Medial opening-wedge high tibial osteotomy (MOWHTO) can be used to correct this malalignment, subsequently alleviating stress on the medial meniscus and reducing pressure in the medial compartment.

The combination of MOWHTO and MMPRT repair has shown improved outcomes in patients with varus alignment.^18,28,29^ Choi et al^5^ reported superior overall healing rates (27.5% vs 10%, respectively) and partial healing rates (60% vs 37.5%, respectively), along with enhanced cartilage regeneration and reduced defect sizes (3.0 vs 4.2 cm^2^, respectively), for combined MOWHTO and MMPRT repair compared with MOWHTO alone. Suh et al^41^ showed that this combined approach effectively preserved the joint space width and reduced medial compartment degeneration. Additionally, Itou et al^15^ documented improved patient-reported outcomes, including Knee injury and Osteoarthritis Outcome Score and Forgotten Joint Score–12 scores, at 15.4 months’ follow-up. These results suggest that concurrent MOWHTO and MMPRT repair promotes better healing and clinical outcomes. In most studies, the target alignment during MOWHTO, with or without MMPRT repair, typically falls within the range of 55% to 62.5%.^5,15,22,28-30^ However, these values were not based on rigorous testing, particularly during MMPRT treatment. The target alignment might differ depending on whether MMPRT repair is performed, as MMPRT repair can restore contact pressure to near-normal levels.^26,32,36,39^ Therefore, experimental data on the biomechanical effects of load redistribution produced by MOWHTO in MMPRT cases will be beneficial.

This study aimed to determine the optimal biomechanical knee alignment during MOWHTO for MMPRT treatment. We hypothesized that medial compartment contact pressure would normalize at different valgus alignments based on the MMPRT status of the knee. Specifically, we predicted that valgus correction of 60% to 65% weightbearing line (WBL) would normalize medial compartment contact pressure without MMPRT repair, whereas valgus correction of 50% to 55% WBL would achieve normalization with MMPRT repair.

## Methods

### Preparation of Cadaveric Specimens

**Figure 1.**
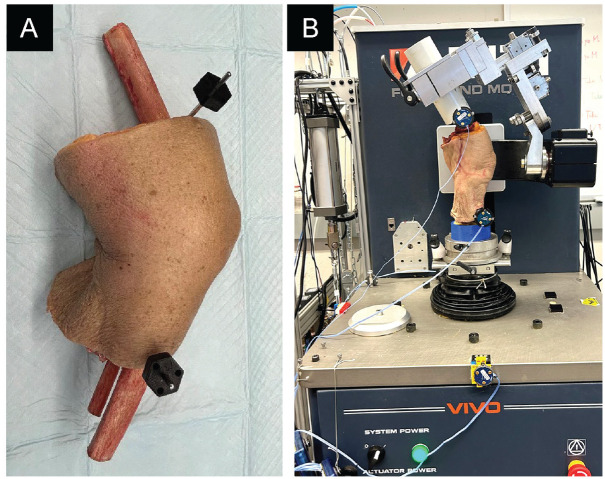
Preparation of the femur and tibia for joint motion simulation. (A) The femur and tibia were cut proximal and distal to the knee joint center, with soft tissue removed. (B) The bones were embedded in pots, fixed, and mounted on a joint motion simulator.

A total of 10 fresh-frozen human cadaveric legs were used for testing. The mean donor age was 61.3 years (range, 33-75 years; 6 male and 4 female). This study was approved by an institutional research ethics board. The specimens were thawed for 24 hours before testing. The femur and tibia were cut 8 inches proximal and 7.5 inches distal to the knee joint center, respectively. All soft tissue 4 inches from the knee joint center was removed from the proximal femur and distal tibia (Figure 1A). The exposed bone was embedded in a polyvinyl chloride pipe/3-dimensionally printed pot and fixed using dental cement (Denstone; Heraeus Kulzer) for mounting on a 6 degrees of freedom joint motion simulator (VIVO; AMTI) (Figure 1B). Optical motion capture markers were rigidly attached to the femur and tibia using 3-dimensionally printed mounts fixed to pins threaded onto the bones. Marker kinematics were tracked using a 6 degrees of freedom optical motion tracking system (Optotrak Certus HD; Northern Digital). Full-length 3-dimensional femoral and tibial models were created from computed tomography images of the cadaveric specimen, coregistered to the motion capture system, and used for visualization of joint kinematics using open-source software for image analysis (3D Slicer; https://www.slicer.org/). This approach enabled the estimation of the coordinates of the femoral head and ankle joint relative to the knee (even though they were transected), allowing for real-time measurements of the mechanical axis passing through the joint.

### Assessment of Tibiofemoral Cartilage Pressure

**Figure 2.**
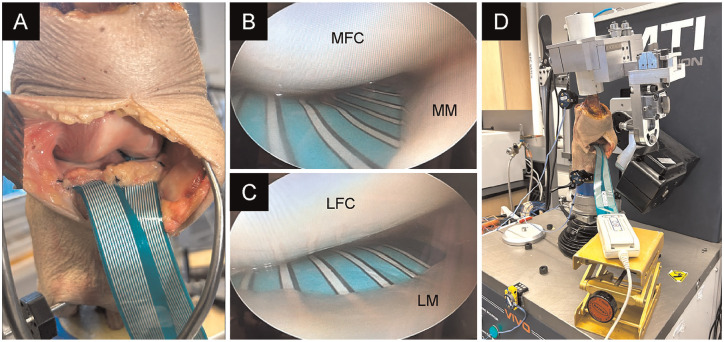
Placement of an electronic pressure sensor. (A) Installation of the pressure sensor beneath the medial meniscus (MM) and lateral meniscus (LM). Confirmation of pressure sensor placement under arthroscopic guidance in the (B) medial and (C) lateral compartments. (D) Mounting the joint motion simulator after pressure sensor placement. LFC, lateral femoral condyle; MFC, medial femoral condyle.

The anterior cruciate, posterior cruciate, and collateral ligaments were intact during testing. Incisions were made in the anterior and posterior portions of the meniscotibial ligament to allow for the placement of a knee pressure sensor (K-Scan System [Model 4011]; Tekscan) under the medial and lateral menisci (Figure 2A). Tekscan sensors have been used in similar studies, demonstrating that they provide valid and reliable measurements of in vitro joint loading.^4,40^ The sensor consists of 2 symmetric matrices, each measuring 24.9 × 40.1 mm, resulting in 273 sensels at a resolution of 27.6 sensels/cm^2^. The sensor could withstand and measure joint contact pressure of up to 3.4 MPa and was calibrated and equilibrated before insertion, following the manufacturer’s guidelines. There were 2 sutures attached to the edge of the sensor and passed in a submeniscal fashion by using the inside-out technique. This allowed the sensor to be pulled along the medial and lateral tibial surfaces under the meniscus until it was placed at the intended position. Sensor placement was confirmed arthroscopically (Figure 2, B and C). A new sensor was used for each specimen. The sensors were submerged in saline solution for 48 hours before testing, which has been reported to minimize gradual deterioration of pressure sensor output caused by liquid exposure during testing. The sensors (and specimens) were kept moist throughout the testing period using normal saline.^16,27^

### Biomechanical Testing

**Figure 3.**
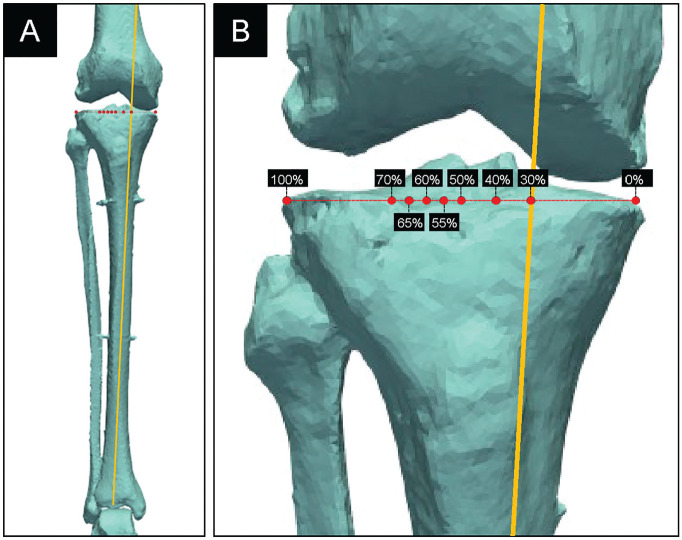
Simulated mechanical alignment. (A) Simulated mechanical alignment (yellow line) connecting the tibial plateau coordinates and the ankle joint center. (B) The tibial plateau coordinates were adjusted to 30%, 40%, 50%, 55%, 60%, 65%, and 70% weightbearing line (WBL), with this image illustrating the simulation at 30% WBL.

Biomechanical testing was performed using a 6 degrees of freedom joint motion simulator (VIVO). After inserting the Tekscan sensor under the medial and lateral menisci, the specimen was mounted on a joint motion simulator (Figure 2D). With the knee in a neutral extended position, the distance of the WBL from the medial border of the tibial plateau was measured and expressed as a percentage of the tibial plateau width, which is referred to as the percentage of the WBL, with 0% indicating that the load passes through the tibial plateau at the medial border and 100% indicating that it passes through the tibial plateau at the lateral border. Clinically, the WBL is adjusted by MOWHTO. However, this approach would have been challenging in this study because of the short length of the transected tibia and the time associated with adjustment of the MOWHTO gap to achieve the desired alignment. Instead, a virtual loading vector through the knee was adjusted using joint motion simulator software, and no actual MOWHTO was performed. Consequently, changes in joint line obliquity and the medial proximal tibial angle were not reproduced. Biomechanically, at the joint surface level, there was no difference between physically changing the alignment of the proximal tibia and changing the applied load trajectory alone. The desired alignment was visualized by marking each point of interest (ie, percentage of the WBL) along the tibial plateau of the 3-dimensional model. The tibial plateau coordinates for WBL measurements were adjusted to 30%, 40%, 50%, 55%, 60%, 65%, and 70% WBL. A loading vector that passed through each of these coordinates and the ankle joint center was defined (Figure 3, A and B). The coordinates of the medial and lateral edges of the tibial plateau were calculated to establish a reference, and the target alignment was determined based on the relative position of the loading vector. Neutral alignment was defined as 50% WBL. After selecting an alignment, a joint load of 700 N, simulating a single-leg stance of a 70-kg person, was applied along the vector for 30 seconds.^10,40^ Medial and lateral compartment pressures were recorded using Tekscan software at 6 frames/s during this time. Pressure mapping software generated a contact pressure map for each condition based on pressure recorded in each cell of a 26 × 22 cell grid. PCP was defined as maximum pressure recorded within the contact area detected by the sensor. In contrast, mean contact pressure (MCP) was calculated as mean pressure within the same area.

### Testing Conditions

**Figure 4.**
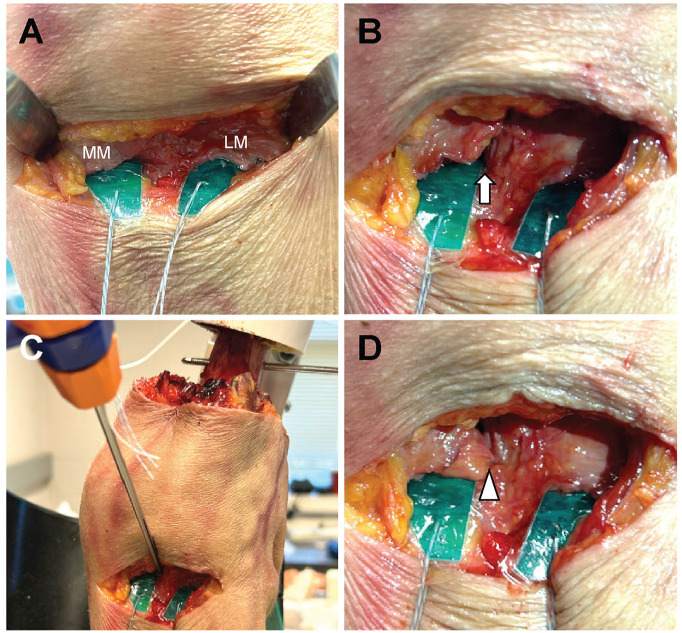
Photographs of a posterior knee. (A) Intact model. (B) Medial meniscus posterior root tear (MMPRT) model, with a white arrow indicating the tear site. (C) Anatomic MMPRT repair using a suture anchor. (D) MMPRT repair model, with a white triangle indicating the repair site. LM, lateral meniscus; MM, medial meniscus.

Each knee underwent 3 testing conditions for the posterior root of the medial meniscus: intact, MMPRT, and repair. In all testing states, the posterior root attachment site of the medial meniscus was directly visualized from the posterior aspect of the knee using a femoral posterior approach (Figure 4A). A posterior root tear of the medial meniscus was created by completely transecting the root directly at the posterior tibial insertion point of the meniscus (Figure 4B). MMPRT repair was performed by visualizing the root attachment at the location of the native root insertion. A TWINFIX suture anchor (Smith & Nephew) was inserted into the attachment, and a mattress suture configuration was formed using the FIRSTPASS MINI suture passer (Smith & Nephew) (Figure 4, C and D). A posterior approach with a suture anchor was used for root repair to ensure optimal access to the root attachment site within the spatial constraints of the joint motion simulator. While transtibial pullout repair is common clinically, studies showing comparable outcomes with suture anchors support its use in this experimental setup.^20,35^ In each condition (intact, MMPRT, and repair), PCP, MCP, and contact area were measured at each simulated alignment (30%, 40%, 50%, 55%, 60%, 65%, and 70% WBL) in both the medial and lateral compartments.

### Statistical Analysis

Statistical analyses were performed using SPSS Statistics for Windows (Version 29.0; IBM). To compare the contact pressure and contact area measurements between the 3 conditions, one-way analysis of variance was conducted, followed by the Tukey post hoc test. The confidence level was set at 95%. The sample size was determined by a power analysis using G*Power (Version 3.1.9.4; Heinrich Heine University Düsseldorf). Based on PCP data from a previous study,^32^ an effect size of 1.06 was calculated. Analysis was performed with a significance level (α) of .05 and statistical power of 80%. These parameters indicated that a minimum of 8 specimens was required to achieve statistical significance.

## Results

### Medial Compartment

#### Peak Contact Pressure

**Figure 5.**
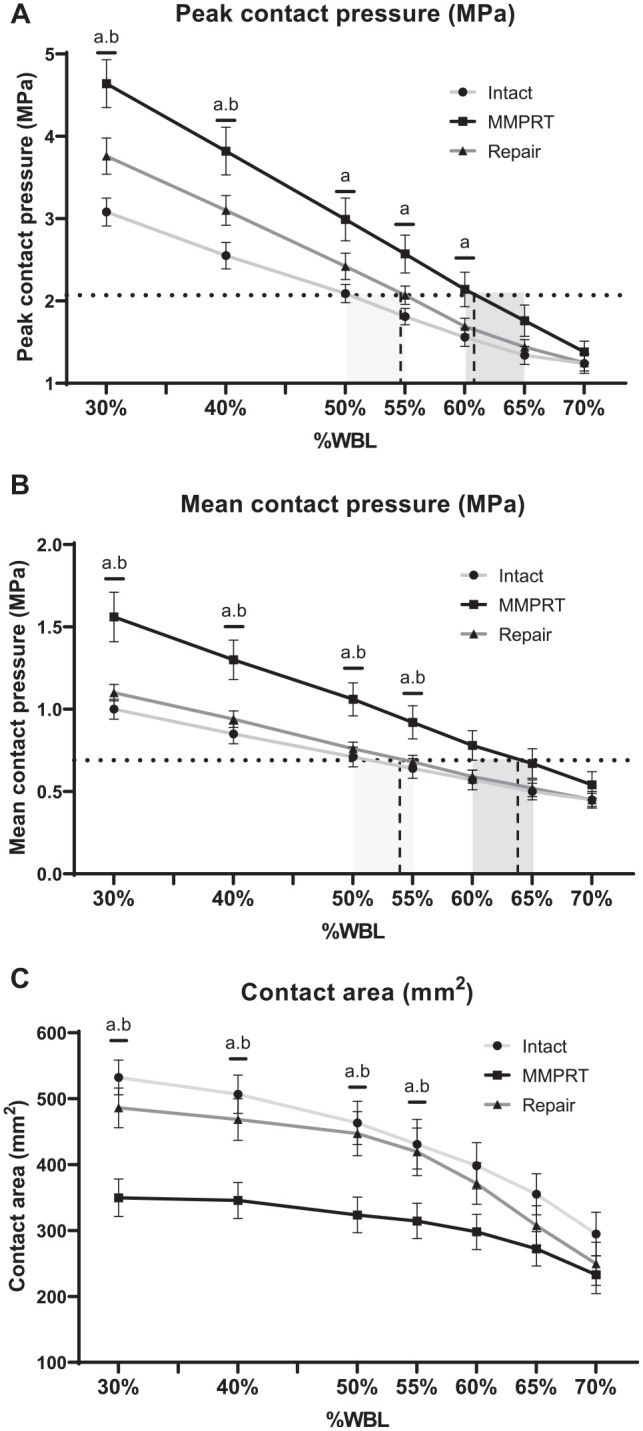
Comparison of contact pressure and contact area between the intact (light gray line), medial meniscus posterior root tear (MMPRT; black line), and repair (dark gray line) conditions at various alignments from 30% to 70% weightbearing line (WBL) in the medial compartment. (A) Peak contact pressure (PCP) at neutral alignment in the intact condition equaled that at 60% to 65% WBL and at 50% to 55% WBL in the MMPRT and repair conditions, respectively. (B) Mean contact pressure (MCP) at neutral alignment in the intact condition equaled that at 60% to 65% WBL and at 50% to 55% WBL in the MMPRT and repair conditions, respectively. The horizontal dashed lines represent (A) PCP or (B) MCP in the intact condition at 50% WBL. The light gray shaded areas indicate the range of alignments, where the dashed lines intersect with the dark gray lines (repair condition). The dark gray shaded areas represent the range of alignments, where the dashed lines intersect with the black lines (MMPRT condition). (C) An MMPRT significantly decreased the contact area compared with the intact condition, whereas MMPRT repair significantly increased the contact area compared with the MMPRT condition, particularly at 30% to 55% WBL. ^a^Statistical significance (*P* < .05) for intact versus MMPRT condition. ^b^Statistical significance (*P* < .05) for MMPRT versus repair condition.

Table 1 and Figure 5A show PCP under each condition for all alignments. In the medial compartment, PCP was increased by 43% in the MMPRT condition (3.0 ± 0.8 MPa) compared with the intact condition (2.1 ± 0.3 MPa) at neutral alignment (*P* = .012); however, after repair (2.4 ± 0.5 MPa), it was not (*P* = .662) (Table 1). Across all alignments, PCP in the MMPRT condition exceeded that in the intact condition, with significant differences at 30% to 60% WBL, peaking at 30% WBL with a 1.5-MPa difference (*P* = .001). In the repair condition, PCP was higher than that in the intact condition across all alignments, but the difference was not significant. PCP gradually increased at varus alignment under all conditions. Moreover, PCP at neutral alignment in the intact condition was comparable with that at 60% to 65% WBL and at 50% to 55% WBL in the MMPRT and repair conditions, respectively (Figure 5A).

**Table 1:** Peak Contact Pressure (MPa) in Medial Compartment^*a*^

	Intact	MMPRT	Repair	*P*
30% WBL	3.1 ± 0.5	4.6 ± 0.9	3.8 ± 0.7	.002^*b*,*c*^
40% WBL	2.6 ± 0.5	3.8 ± 0.9	3.1 ± 0.5	.002^*b*,*c*^
50% WBL	2.1 ± 0.3	3.0 ± 0.8	2.4 ± 0.5	.013^*b*^
55% WBL	1.8 ± 0.3	2.6 ± 0.7	2.1 ± 0.3	.011^*b*^
60% WBL	1.6 ± 0.3	2.1 ± 0.6	1.7 ± 0.3	.032^*b*^
65% WBL	1.3 ± 0.3	1.8 ± 0.6	1.4 ± 0.3	.208
70% WBL	1.2 ± 0.3	1.4 ± 0.4	1.3 ± 0.3	.844

a Data are presented as mean ± SD. MMPRT, medial meniscus posterior root tear; WBL, weightbearing line.

b Statistically significant (*P* < .05): intact versus MMPRT.

c Statistically significant (*P* < .05): MMPRT versus repair.

#### Mean Contact Pressure

Table 2 and Figure 5B show MCP for each condition across all the alignments. At neutral alignment, MCP was increased significantly in the MMPRT condition (1.1 ± 0.3 MPa) compared with that in the intact condition (0.7 ± 0.2 MPa) (*P* = .006) and repair condition (0.8 ± 0.1 MPa) (*P* = .015) (Table 2). MCP was significantly higher in the MMPRT condition than in the intact and repair conditions at 30% to 55% WBL (*P* < .05). MCP in the repair condition was similar to or higher than that in the intact condition across all alignments; however, the difference was not statistically significant (*P* > .05). MCP gradually increased at varus alignment under all the conditions. No significant differences were observed between the intact condition at 50% WBL and the MMPRT condition at 60% or 65% WBL (*P* = .281 and *P* = .638, respectively), but significant differences were observed at 55% and 70% WBL (*P* = .008 and *P* = .013, respectively). Similarly, no significant differences were observed between the intact condition at 50% WBL and the repair condition at 50% or 55% WBL (*P* = .129 and *P* = .394, respectively), whereas a significant difference was observed at 60% WBL (*P* = .019). At neutral alignment, MCP in the intact condition corresponded with that at 60% to 65% WBL and at 50% to 55% WBL in the MMPRT and repair conditions, respectively (Figure 5B).

**Table 2:** Mean Contact Pressure (MPa) in Medial Compartment^*a*^

	Intact	MMPRT	Repair	*P*
30% WBL	1.0 ± 0.2	1.6 ± 0.4	1.1 ± 0.2	<.001^*b*,*c*^
40% WBL	0.9 ± 0.2	1.3 ± 0.4	0.9 ± 0.1	.001^*b*,*c*^
50% WBL	0.7 ± 0.2	1.1 ± 0.3	0.8 ± 0.1	.006^*b*,*c*^
55% WBL	0.6 ± 0.2	0.9 ± 0.3	0.7 ± 0.1	.011^*b*,*c*^
60% WBL	0.6 ± 0.2	0.8 ± 0.3	0.6 ± 0.1	.084
65% WBL	0.5 ± 0.1	0.7 ± 0.3	0.5 ± 0.1	.143
70% WBL	0.5 ± 0.1	0.5 ± 0.2	0.5 ± 0.1	.331

a Data are presented as mean ± SD. MMPRT, medial meniscus posterior root tear; WBL, weightbearing line.

b Statistically significant (*P* < .05): intact versus MMPRT.

c Statistically significant (*P* < .05): MMPRT versus repair.

#### Contact Area

Figure 5C shows the contact area for each condition across all alignments. In the MMPRT condition, the contact area was significantly decreased by 30% (323.7 ± 80.8 mm^2^) compared with the intact condition (463.2 ± 98.0 mm^2^) (*P* < .001) at neutral alignment. In contrast, the contact area was significantly increased in the repair condition (447.1 ± 100.2 mm^2^) compared with that in the MMPRT condition (*P* = .003). The contact area was consistently lower in the MMPRT condition than that in the intact condition, with a significant decrease at 30% to 55% WBL (*P* < .05). However, the contact area in the repair condition was higher than that in the MMPRT condition at all alignments, with significant differences at 30% to 55% WBL (*P* < .05). The contact area in the repair condition was generally lower than that in the intact condition at most alignments. However, these differences were not statistically significant, suggesting that the repair technique maintained contact characteristics similar to those of the intact state.

### Lateral Compartment

**Figure 6.**
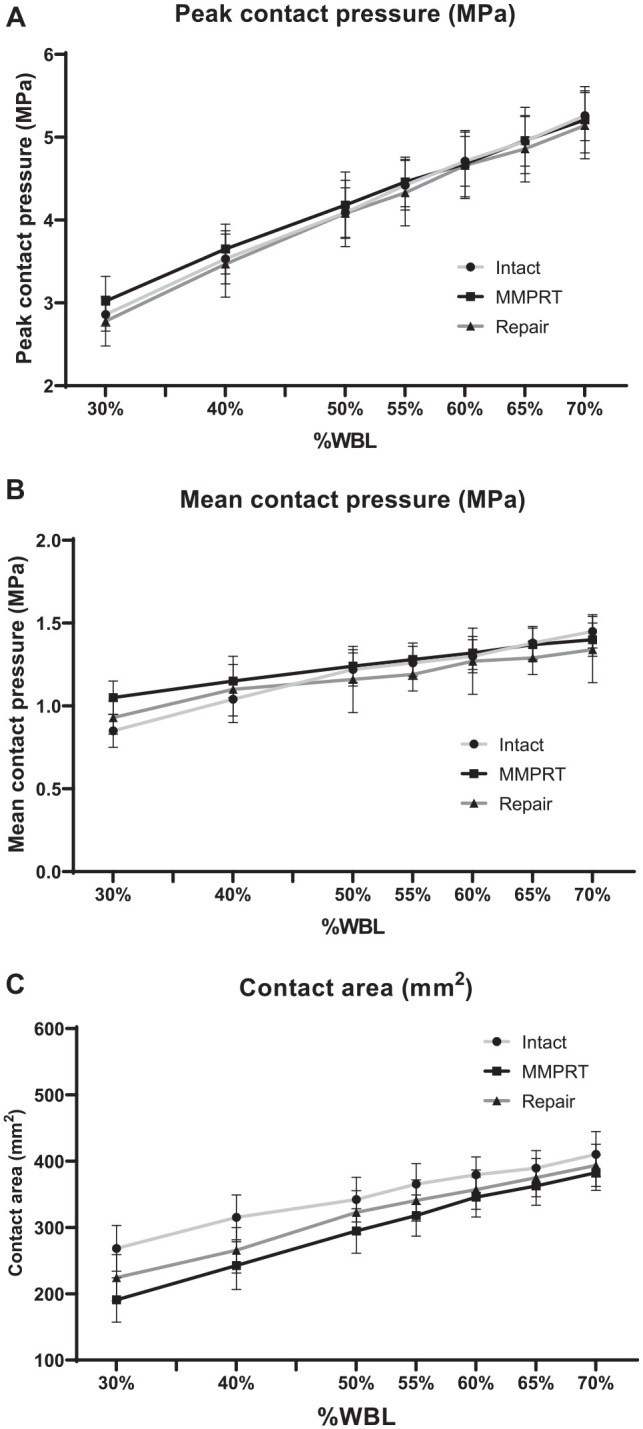
Comparison of contact pressure and contact area between the intact (light gray line), medial meniscus posterior root tear (MMPRT; black line), and repair (dark gray line) conditions at various alignments from 30% to 70% weightbearing line (WBL) in the lateral compartment. (A) Peak contact pressure, (B) mean contact pressure, and (C) contact area.

Figure 6 shows PCP, MCP, and contact area in the lateral compartment. At neutral alignment in the intact condition, PCP, MCP, and contact area measured 4.2 ± 0.8 MPa, 1.2 ± 0.4 MPa, and 342.0 ± 106.2 mm^2^, respectively. PCP in the intact condition was gradually increased from 2.9 ± 0.7 MPa at 30% WBL to 5.4 ± 0.8 MPa at 70% WBL, with no significant differences among the 3 conditions at all alignments (Figure 6A). MCP and contact area followed similar trends to PCP, also showing no significant differences among the conditions across all alignments (Figure 6, B and C).

## Discussion

The principal findings of this study demonstrated that medial compartment PCP and MCP at neutral alignment in the intact condition corresponded to those observed at 60% to 65% WBL and at 50% to 55% WBL in the MMPRT and repair conditions, respectively. These findings provide biomechanical guidance for determination of the target alignment in knees with MMPRTs based on the meniscal status.

Several biomechanical studies have previously been conducted on MMPRTs.^4,19,26^ Marzo and Gurske-DePerio^32^ found that after MMPRTs, PCP increased significantly by approximately 32% (from 3841 to 5084 kPa), whereas the contact area showed a significant decrease (from 594 to 474 mm^2^). Similarly, Allaire et al^1^ reported that PCP on the medial side increased by 25%, which is equivalent to complete medial meniscectomy. In our study, 43% and 57% increases in PCP and MCP, respectively, were observed at neutral alignment, yielding results consistent with those of previous research.^1,32^ This increase in contact pressure and reduction in the contact area due to an MMPRT may contribute to the progression of medial compartment arthrosis and functional deterioration. Notably, our findings suggest that the significant increase in contact pressure associated with an MMPRT compared with an intact knee was amplified as varus alignment increased. Specifically, the maximum difference in PCP was observed at 30% WBL, with an increase of 1.5 MPa, as well as in MCP by 0.6 MPa. These results underscore the exacerbation of contact pressure due to an MMPRT at increased varus alignment, highlighting the importance of considering lower limb realignment in MMPRT treatment.

To the best of our knowledge, to date, only a few studies have examined the biomechanical effects of alignment correction in MMPRTs. Many studies have referred to the “Fujisawa point” as the target alignment for knees with MMPRTs based on an article published by Fujisawa et al,^12^ which determined that ideal mechanical axis alignment during osteotomy, exceeding 30% to 40% lateral to the midpoint, promotes arthroscopic healing of the meniscus and cartilage. However, it is worth noting that this target alignment is primarily intended for the treatment of medial osteoarthritis, raising questions regarding its direct applicability to MMPRT treatment. Park et al^38^ investigated the biomechanical effects of MMPRT repair during MOWHTO at fixed valgus angles of 0°, 5°, and 10°. Their findings demonstrated that MMPRT repair reduces contact pressure, regardless of valgus correction, underscoring its role in protecting the medial compartment. Although their study did not incorporate clinically relevant alignment adjustments, our study analyzed a broader and more clinically applicable range of alignments (30%-70% WBL), using a combination of 5% and 10% increments, enabling a more precise evaluation.

In our study, PCP and MCP were normalized to 60% to 65% WBL in the MMPRT condition compared with the intact condition at neutral alignment. Several factors, including older age, preoperative varus alignment, and severe preoperative chondral lesions, have been reported as risk factors for clinical failure after MMPRT repair.^6,7,17^ If an MMPRT is irreparable or if a rerupture is anticipated after root repair, HTO may be required to normalize contact pressure in the medial compartment and mitigate osteoarthritis progression. Recent studies have reported the use of a target alignment of 62% to 62.5% during MOWHTO for MMPRTs, finding successful clinical and radiographic outcomes in the short term.^5,14,42^ In our study, without MMPRT repair, 60% to 65% valgus correction normalized medial compartment contact pressure. The alignment range of 60% to 65% WBL in our study is similar to the optimal range indicated in previous literature, thereby bolstering the validity of the proposed target alignment in prior clinical studies.

Conversely, our study further supports the necessity of achieving 50% to 55% valgus correction to normalize medial compartment contact pressure in the repair condition. While 50% WBL alone was insufficient to fully restore medial compartment contact pressure to the levels observed in intact knees, adjustments within the range of 50% to 55% WBL successfully normalized medial compartment contact pressure, highlighting the importance of this target range for achieving biomechanical balance while minimizing the risk of lateral compartment overloading. The report by Feucht et al,^11^ which suggests 50% to 55% correction for medial meniscal transplantation without signs of osteoarthritis, aligns closely with our findings. MMPRT repair has been shown to be effective in restoring joint contact biomechanics. However, it was intriguing to observe that even repairing MMPRTs at neutral alignment still resulted in approximately 14% higher PCP than that in intact knees, failing to return contact pressure levels to normal (2.1 MPa in intact knees vs 2.4 MPa in repaired knees). Previous studies have reported similar findings, indicating that while anatomic repair normalizes, it does not fully restore healthy contact pressure levels. For example, LaPrade et al^26^ revealed a 26% and 53% increase in PCP with anatomic and nonanatomic repair, respectively, compared with intact knees. Similarly, Padalecki et al^36^ observed a 19% increase in PCP even after MMPRT repair compared with intact knees. These results reveal that while clinical outcomes may initially improve after MMPRT repair, long-term issues such as poor meniscal healing, meniscal extrusion progression, and osteoarthritis development may persist, underscoring the limitations of MMPRT repair alone.^7,33^

The findings of this study emphasize the importance of adjusting the target alignment based on the meniscal and cartilage status. Under the assumption of successful meniscal repair, our results suggest that 50% to 55% correction is sufficient for cases with minimal degenerative changes and successful repair. However, in cases in which the meniscus is inadequately repaired, or a retear is anticipated, 60% to 65% correction may be required to lower medial compartment pressure and mitigate osteoarthritis progression. While consistently pursuing 60% to 65% correction lowers medial compartment pressure and may be necessary if a retear or suboptimal healing of the meniscus is anticipated, it may not be the optimal strategy for all patients. In knees with minimal degenerative changes in the medial compartment or successful meniscal repair, less correction of 50% to 55% may sufficiently restore pressure while minimizing the risk of overloading the lateral compartment. This approach enables a tailored alignment based on the patient’s cartilage or meniscal status.

Interestingly, even in cases of valgus alignment, MMPRT repair reduced medial compartment contact pressure. This finding aligns with recent biomechanical studies demonstrating that MMPRT repair during MOWHTO consistently reduces contact pressure and increases the contact area in the knee, regardless of the degree of valgus correction.^38^ Several clinical studies have compared the outcomes of MOWHTO alone (with a target alignment of 62%-62.5%) with those of MOWHTO combined with MMPRT repair.^18,28,29^ Although MMPRT repair has shown improvements in arthroscopic outcomes, such as tear healing and cartilage regeneration, it has not significantly enhanced short-term clinical or radiological outcomes.^18,28,29^ Although the superiority of MMPRT repair in conjunction with MOWHTO has not been definitively established, our study suggests that when repairable MMPRTs are present, MMPRT repair during MOWHTO may reduce contact pressure, potentially preventing osteoarthritis progression and contributing to improved long-term clinical outcomes.

In the lateral compartment, each condition demonstrated a similar trend across various alignments. Specifically, contact pressure and contact area increased at greater valgus alignment, and no significant differences were observed between the 3 conditions. This suggests that the presence or absence of an MMPRT and its repair has a minimal effect in the lateral compartment. Lee et al^30^ reported that excessive valgus correction beyond 67% resulted in poor postoperative patient-reported outcomes. Excessive valgus correction is believed to increase the medial proximal tibial angle and joint line obliquity, leading to increased shear stress and osteoarthritis progression in the patellofemoral compartment; therefore, this should be avoided.^34^ Moreover, Martay et al^31^ demonstrated that correcting the WBL to a range between 62% and 65% (3.4°-4.6° valgus) further reduced medial stress but posed a risk of damaging lateral compartment tissue. Our study supports these findings, showing that at 65% WBL, PCP in the lateral compartment was 3.9 times greater than that in the medial compartment, increasing by a factor of 4.5 times at 70% WBL in the MMPRT condition. This suggests that correction exceeding 65% should be approached with caution because of increased loading in the lateral compartment, which may lead to cartilage degradation or lateral meniscal tearing. Therefore, the range of 60% to 65% obtained in our study appears to be a reasonable target from the perspective of minimizing the lateral compartment load.

### Limitations

Despite the significant insights revealed in our study, some limitations must be considered when interpreting these results. First, the cadaveric specimens used in this study did not replicate early osteoarthritis changes, meniscal extrusion, or the degenerative nature of meniscal tissue, which are common in knees with MMPRTs. Moreover, meniscal extrusion in MMPRTs often results from meniscotibial ligament damage or stretching, which was not completely re-created in this model.^23,37^ Second, this was a time-zero study and did not assess the ability of the meniscus to heal. Third, actual MOWHTO was not performed, and the effects of joint line obliquity and the medial proximal tibial angle were not considered. Additionally, each percentage of the WBL was calculated geometrically using tibial plateau dimensions without MOWHTO, representing a methodological limitation. Fourth, the simulated mechanical axis used in this study was not a true mechanical axis connecting the hip and ankle centers. Instead, the mechanical axis was defined as the loading vector from the tibial plateau to the center of the ankle. The ankle center coordinates remained fixed, while the tibial plateau coordinates were modified to simulate osteotomy. By defining the loading vector origin with respect to the tibial plateau, we were able to minimize shifting of the mechanical axis when applying physiologically relevant joint loads. Finally, this study classified alignment changes in discrete 5% increments of the WBL. Although this approach allowed for practical testing within the experimental setup, a finer classification (eg, 1% increments) could provide a more precise understanding of the relationship between alignment and contact pressure. However, testing at such small increments is limited by the extended time required for each experiment and the potential sensitivity degradation of the Tekscan sensor over time. Although this study provides biomechanical insights, it is based on a simulated osteotomy model and lacks direct clinical validation. Furthermore, knee contact pressure was not evaluated under flexion or dynamic gait conditions, limiting the generalizability of the findings to clinical practice. Future research is needed to develop this biomechanical study and to confirm whether the proposed alignment targets lead to improved clinical outcomes.

## Conclusion

Medial compartment contact pressure at neutral alignment in the intact condition was similar to that at 60% to 65% WBL in the MMPRT condition and at 50% to 55% WBL in the repair condition, indicating optimal biomechanical alignment targets for MOWHTO in patients with MMPRTs.
